# The Prevalence and Associated Factors of Pes Planus Among Albanian Secondary School Students: A Cross-Sectional Study

**DOI:** 10.7759/cureus.90766

**Published:** 2025-08-22

**Authors:** Vjollca Shpata, Andromeda Lalaj, Klejda Tani, Ajsel Oseku, Xhensila Prendushi, Albana Shabani

**Affiliations:** 1 Faculty of Rehabilitation Sciences, Sports University of Tirana, Tirana, ALB; 2 Faculty of Medicine, University of Medicine, Tirana, ALB; 3 Faculty of Medical Technical Sciences, University of Medicine, Tirana, ALB

**Keywords:** adolescents, body mass index, pes planus, prevalence, scoliosis

## Abstract

Background: Pes planus (flat feet) is a common postural condition among children and adolescents, characterised by a collapsed medial longitudinal arch. Its aetiology is multifactorial, and persistence beyond childhood may lead to long-term musculoskeletal complications.

Objective: This study aimed to determine the prevalence of pes planus among secondary school students in Tirana, Albania, and assess possible associations with gender, age, body mass index (BMI), height, weight, and spinal deformities such as scoliosis.

Methods: A cross-sectional study was conducted between January and May 2024 in nine public schools in Tirana. A total of 2020 students aged 10-15 years participated. Assessment tools included the Navicular Drop Test, longitudinal ankle angle, Trendelenburg test, Adams test, and measurements with a scoliometer for scoliosis. BMI percentiles were classified according to the World Health Organization growth charts for individuals aged 5-19 years. Statistical analysis was performed using IBM SPSS Statistics for Windows, Version 20 (Released 2015; IBM Corp., Armonk, New York, United States), with significance set at p < 0.05.

Results: Pes planus was identified in 474 students (204 female students and 270 male students), representing 23.46% of the participants. Male students showed a significantly higher prevalence at 26.16% (n = 270) compared to female students, who had a prevalence of 20.65% (n = 204) (χ²(1) = 8.544, DF = 1, φ = 0.065, p = 0.0035). Male students also had greater odds of having pes planus compared to female students (OR: 1.36, 95% CI: 1.01-1.68, p = 0.003). No statistically significant association was found between pes planus and BMI, body height, scoliosis, or participation in physical activity. The prevalence of pes planus was higher in older age groups, reaching a peak at 13 years, with 26.78% (n = 109), and at 14-15 years, with 25.58% (n = 76).

Conclusion: Pes planus is prevalent among Albanian adolescents, particularly in the male population. Early screening during school years is advisable to prevent potential long-term complications. No significant correlations were found between pes planus and weight, height, or the presence of scoliosis.

## Introduction

Pes planus (flat feet) is a common condition characterised by a collapsed medial longitudinal arch, heel valgus, and medial talar prominence [1]. Numerous studies have identified a relatively high prevalence of flat feet, ranging from 10.27% to 17.1% [1,2]. In 2006, a study by Pfeiffer and colleagues observed an even higher prevalence of 44% among preschool-aged children [3].

Racial and ethnic differences in foot disorders and foot types have been well documented [4]. In the Mediterranean region, research in Greece has characterised the foot morphology of children and adolescents [5], while a survey in Southern Italy found a 9.35% prevalence of flat feet among individuals aged 10-20 years [6]. Although geographically close, Albania has distinct nutritional and cultural traits (related to physical activity or sedentary behaviour, and footwear habits) that may influence pes planus prevalence [7]. Notably, epidemiological data on pes planus in adolescents remain limited across the wider Western Balkan region.

Arch collapse alters lower limb biomechanics, increasing the risk of pain, injury, and disability [7]. Sometimes, the deformity develops into a painful, inflexible shape that significantly impairs function; however, more often, it remains asymptomatic and resolves on its own within the first 10 years of life [6]. The main issues associated with pes planus include congenital vertical talus, tarsal coalition, equinus deformity of the foot, tibial torsional deformity, ligamentous laxity, and the presence of the accessory navicular bone. Moreover, adult flat feet can be classified as either acquired or residual flat feet deformity resulting from a developmental origin. Traumatic deformities, ruptured plantar fascia, Charcot's foot, posterior tibial tendon dysfunction, midfoot laxity, forefoot abduction, external hindfoot rotation, talus subluxation, tight triceps surae or isolated gastrocnemius, and neuromuscular imbalance (polio, cerebral palsy, closed head injury, or following a cerebrovascular accident) are all linked to acquired flat feet [8]. Although studies have shown that flat feet often resolve spontaneously during the first decade of life [9], some research indicates that flat feet persist with increasing age [10,11]. Among individuals aged 18-25 years, a 29% prevalence of pes planus was reported, reinforcing evidence that flat feet may not always resolve with age [11]. A recent study revealed that out of 2994 participants aged 36-98 years, pes planus was present in at least one foot for 74% of the study population, demonstrating that arch height decreases with age [10]. In these individuals, hallux valgus or hallux rigidus was also observed, indicating an increased probability that flat feet will lead to other foot disorders. Consequently, to avert abnormalities associated with flat feet, such as crowding of the lesser toes, medial navicular prominence, rear foot eversion, and secondary hallux valgus, along with stretching of the tibialis posterior tendon and the plantar calcaneonavicular ligament, early foot screening is essential [12].

Early detection of pes planus during periods of rapid growth, such as adolescence, is essential, as undiagnosed or untreated deformities can lead to long-term complications.

Individuals with moderate to severe pes planus experienced nearly twice the incidence of anterior knee pain and occasional low back pain [13]. Untreated pes planus can lead to significant long-term musculoskeletal issues. Altered foot biomechanics increase medial loading of the knee, which has been linked to impaired knee function and a higher risk of developing medial compartment knee osteoarthritis [14]. Changes in foot posture may also impair static balance and reduce proprioceptive acuity at the ankle and knee, potentially raising the risk of falls and lower-limb injuries [15]. Over time, these biomechanical changes can contribute to abnormal gait patterns, increased energy expenditure during walking, and compensatory strain on proximal joints, including the hips and spine [16].

Although many epidemiological studies have been conducted across different populations, data on the prevalence and associated factors of pes planus among Albanian youth are currently unavailable.

The primary aim of this study is to determine the prevalence of pes planus among secondary school students aged 10 to 15 years in Tirana, Albania. A secondary aim is to investigate its possible associations with age, gender, body mass index (BMI), height, weight, and spinal deformities such as scoliosis.

## Materials and methods

A cross-sectional study was conducted from January to May 2024 in nine public secondary schools across Tirana, Albania.

The sample size was calculated using the single population proportion formula [17], assuming a pes planus prevalence of 26.62% [18], a 95% confidence level, and a 3% margin of error, resulting in a minimum of 833 participants. The study involved 2020 students aged 10 to 15 years, corresponding to grades 5 to 9. The final sample of 2,020 children, representing approximately 8% of all 10-15-year-old students in urban Tirana, exceeded this requirement. With 80% of students attending public schools, the exclusive inclusion of public institutions is unlikely to have introduced selection bias.

The consent form was given to the parents or legal guardians of the students one week before the scheduled measurement sessions.

Participants were eligible if they attended scheduled physical education classes, had obtained written informed consent from a parent or guardian, and provided assent to participate.

The study complied with the ethical standards outlined in the Declaration of Helsinki, and the Committee for Research, Projects, and Scientific Publications, established by the Academic Senate of the Sports University of Tirana, approved the study protocol. Permission was also granted by the Ministry of Education and Sport. All assessments were conducted anonymously to protect participant confidentiality.

Anthropometric and clinical assessments

During scheduled school visits in physical education classes, experienced physiotherapists carried out anthropometric assessments, which included measurements of weight and height. Body weight and height were measured using calibrated equipment, and BMI was calculated as kg/m². BMI percentiles were classified according to the World Health Organisation growth charts for individuals aged 5-19 years [19]. Participants were categorised as overweight (85th-94th percentile), obese (≥95th percentile), or severely obese (≥120% of the 95th percentile or ≥35 kg/m²).

The survey participants were divided into five age groups in one-year intervals (10, 11, 12, 13 years); however, the 14- and 15-year-old groups were combined due to the limited number of 15-year-old participants.

Physiotherapists evaluated foot deformities using the following measurements as valid tests related to pes planus: (i) The Navicular Drop Index measures the vertical displacement of the navicular tuberosity between non-weight-bearing (seated) and weight-bearing (standing) positions. A drop greater than 10 mm was considered indicative of pes planus [20] and (ii) longitudinal ankle angle (LAA): The goniometer was placed over the navicular tuberosity, with the arms aligned to the medial malleolus and the head of the first metatarsal. The angle between these lines was measured in degrees, and an angle lower than 131° was used to classify pes planus [21].

Additionally, the Trendelenburg Test (observing the pelvis position while the student stood on one leg) was used to assess pelvic stability, considering that the gluteus medius muscles are crucial during walking and act as pelvic stabilisers; foot deformity can contribute to muscle weakness and gait [22]. The Trendelenburg sign is considered positive when, during a single-leg stance on either leg, the patient cannot maintain the pelvis level with the floor.

Spinal deformities were screened using the Adams Forward Bend Test. For suspected cases of scoliosis (where the Adams test was positive), a scoliometer was utilised. A scoliometer reading exceeding 7° was regarded as indicative of scoliosis [9]. The assessment for scoliosis was carried out because adolescents with scoliosis and pes planus have been observed to experience increased plantar pressure in the midfoot and posterior arch of the foot compared to individuals with a normal foot shape, suggesting that pes planus may influence the distribution of forces during stance and gait, potentially contributing to the development or progression of scoliosis [23].

To assess whether pes planus restricts participation in daily physical activities, the students answered questions about whether they engage in sports or physical activity after school, and how many hours per week they spend on physical activity, including the hours of physical education classes.

Statistical analysis

Descriptive statistics were used to summarise participant characteristics. Continuous variables were presented as means and standard deviations, while categorical variables were reported as frequencies and percentages. Group comparisons employed Student’s t-test for continuous data and the chi-square test for categorical data. Univariate regression analysis examined variables associated with pes planus, such as gender, age, BMI, height, weight, spinal deformities (such as scoliosis), positive Trendelenburg sign, and physical activity level.

Statistical analyses were performed using IBM SPSS Statistics for Windows, Version 20 (Released 2015; IBM Corp., Armonk, New York, United States). A p-value of less than 0.05 was regarded as statistically significant.

## Results

A total of 2,020 students aged 10 to 15 years participated in the study, with a mean age of 11.98 ± 1.25 years; 48.9% (n = 987) were female.

Prevalence of pes planus

Pes planus was identified in 474 participants (204 female students and 270 male students), indicating a prevalence of 23.46% (95% CI: 21.6%-25.3%). The condition was more common among male students (26.16%) than female students (20.65%), with a significant difference (χ²(1) = 8.544, DF = 1, φ = 0.065, p = 0.0035). Male students showed higher odds of having pes planus compared to female students (OR: 1.36, 95% CI: 1.01-1.68, p = 0.003).

Table 1 shows that the highest prevalence of pes planus was among 13-year-olds at 26.78% (n = 109), followed by 14-15-year-olds at 25.58% (n = 76). The lowest prevalence was observed at age 10 at 20.64% (n = 45). Table 2 presents data on the study population, categorised by whether they have pes planus or a normal foot position.

**Table 1:** The prevalence of pes planus across different age groups

Age Groups in Years	Neutral Number (%)	Pes Planus Number (%)	Total Number (%)
10	173 (79.36%)	45 (20.64%)	218 (10.79%)
11	470 (77.81%)	134 (22.18%)	604 (29.90%)
12	384 (77.73%)	110 (22.27%)	494 (24.45%)
13	298 (73.22%)	109 (26.78%)	407 (20.15%)
14 - 15	221 (74.41%)	76 (25.58%)	297 (14.70%)
Total	1546 (76.5%)	474 (23.5%)	2020 (100%)

**Table 2:** Study population data according to the presence of pes planus or the neutral position of the feet

Study Participants' Data	Pes Planus	Neutral Foot	Total
Female, No (%)	204 (43.04%)	784 (50.71%)	988 (48.9%)
Male, No (%)	270 (56.96%)	762 (49.61%)	1032 (51.1%)
Body weight (kg) (Mean ± SD)	49.75±12.69	50.54±13.29	50.35±13.15
Height in cm (Mean ± SD)	155.29± 10.52	155.47±9.85	155.44±10.01
Body mass index (kg/m^2^) (Mean ± SD)	20.24±4.01	20.64±4.44	20.55±4.35
Presence of scoliosis, No (%)	13 (2.74%)	50 (3.26%)	63 (3.12%)
Positive Trendelenburg test, No (%)	264 (55.69%)	889 (57.88%)	1152 (71.88%)
Participate in sports, No (%)	149 (31.43%)	526 (34.24%)	675 (33.42%)
Do not participate in sports, No (%)	325 (68.56%)	1020 (66.41%)	1345 (66.58%)

Associations with anthropometric variables and scoliosis

No statistically significant associations were observed between pes planus and either height (p = 0.61) or weight (p = 0.48). When analysed by BMI categories, the highest prevalence of pes planus was among overweight participants at 25.59% (n = 108), followed by those with normal body weight at 23.82% (n = 303), and those classified as obese at 21.51% (n = 54), as shown in Table 3. However, these differences were not statistically significant (p > 0.05).

**Table 3:** Prevalence of pes planus based on body weight status

Foot position	Average Weight	Obese	Overweight	Severely Obese
Neutral position of the foot	969 (76.18%)	197 (78.49%)	314 (74.41%)	66 (88%)
Pes planus	303 (23.82%)	54 (21.51%)	108 (25.59%)	9 (12%)

Scoliosis was detected in 63 students (3.11%) based on scoliometer readings exceeding 7°. Among these students, 13 students (20.63% of those with scoliosis) also had pes planus, whereas in those without scoliosis, the prevalence of pes planus was 23.56% (461 students), showing a non-significant difference (χ²(1) = 0.290, DF = 1, p = 0.59, φ= 0.012). Therefore, no link was found between pes planus and scoliosis.

Similarly, no significant association was found between a positive Trendelenburg test and the presence of pes planus. Two hundred and sixty-three (55.48%) students with pes planus exhibited a positive Trendelenburg sign, whereas in the group with neutral foot position, 890 students (57.57%) showed a positive sign (χ²(1) = 0.615, DF = 1, p = 0.43, φ = 0.017). The presence of pes planus did not influence hip abductor strength or their ability to stabilize the pelvis any more than in participants with a neutral foot position.

There was no observed difference in physical activity levels between participants with and without pes planus. Students with a neutral foot position spend 6.64 ± 4.16 hours per week on physical activity, compared with 6.66 ± 4.13 hours per week for students with pes planus, p = 0.92.

34.24% of students with neutral feet position were engaged in sports, and 31.43% of students with pes planus, showing no difference in sport participation between the two groups.

**Figure 1:**
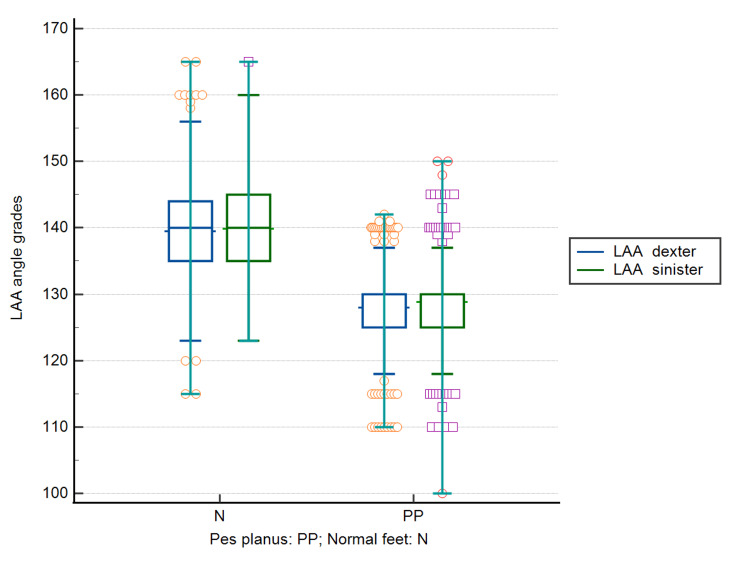
Longitudinal ankle angle (LAA) values based on the presence or absence of pes planus

Figure 1 shows that participants with pes planus had a significantly lower LAA than those with a neutral foot posture, in both non-weight-bearing and weight-bearing positions. Mean values of LLA dexter were 139.51 ± 6.22 degrees in participants with a neutral foot position and 128.01 ± 5.73 degrees in participants with pes planus (t-test: difference -11.5, 95% CI: -12.13 to -10.87, DF = 2018, p < 0.0001).

Mean values of LLA sinister were 139.88 ± 5.84 degrees in participants with a neutral foot position, and 128.81 ± 5.86 degrees in participants with pes planus (t-test: difference -11.7, 95% CI: -11.67 to -10.46, DF = 2018, p < 0.0001).

## Discussion

This study found that among 2,020 students, 474 exhibited pes planus, resulting in a prevalence of 23.5% among secondary school pupils aged 10 to 15 years in Tirana, Albania.

This rate aligns with existing literature reporting figures between 10% and 30% in adolescent populations [7], confirming that pes planus is a common postural condition during this developmental stage with potential long-term biomechanical and musculoskeletal consequences.

Notably, among Albanian adolescents, male participants showed a significantly higher prevalence (26.16%) compared to female participants (20.65%), consistent with previous studies in other populations [7]. This finding supports earlier research indicating that medial arch development in males progresses more slowly during adolescence, possibly due to hormonal and biomechanical differences affecting musculoskeletal maturation. During adolescence, testosterone-driven increases in bone and muscle mass, along with rapid gains in stature and body weight, can increase mechanical stress on the foot. This disproportionate growth has been associated with changes in midfoot geometry and a higher risk of lowering the medial longitudinal arch [9]. Moreover, the physiological transition of foot morphology from low-arched to normal-arched types occurs at an earlier age in girls than in boys [5], with flatfoot tending to persist longer in boys than in girls. A series of additional studies on adult populations has also associated men with a greater predisposition to pes planus [1,24].

However, some studies have reported differing gender distributions, with a higher prevalence in the female population [18], emphasising the need for further gender-specific research, along with consideration of environmental and lifestyle factors.

Evidence indicates that the prevalence of flat feet declines with age [6,22]. The findings of Stavlas and colleagues demonstrate that foot morphology undergoes significant developmental changes between six and 17 years of age, with a progressive reduction in flatfoot prevalence as children mature [5].

However, in our study, such an approach is not correlational, as the ages of 13 (26.78%) and 14-15 (25.58%) show a higher prevalence than 10-year-olds (20.64%), similar to some studies that did not observe any relation between flat feet and age [18].

Although some literature associates obesity and higher BMI with pes planus [18], our study did not find a statistically significant link between BMI and the presence of flat feet. While the overweight group showed a marginally higher prevalence of pes planus (25.59%) compared to the average body weight group (23.82%), participants who were obese or severely obese had a lower prevalence of pes planus (21.51% and 12%, respectively) compared to their counterparts with an average body weight. Our results contrast with studies indicating a 2.5 times greater risk of flat feet in overweight or obese children compared to those of normal weight [25]. Increased body mass imposes greater load-bearing demands on the developing foot, which may predispose to valgus alignment and the progressive collapse of the medial longitudinal arch. However, the prevalence of pes planus among overweight individuals in our population was higher than that of the obese group, which is inconsistent with other studies [26]. These findings emphasise the complex and multifactorial relationship between body composition and pes planus, highlighting the need for further research.

Furthermore, our study found no link between body height and the presence of pes planus, in contrast to studies that have demonstrated a decreased risk of pes planus associated with high body height [26].

Pes planus occurs when the longitudinal and/or medial arches of the foot are flat or lowered, causing the entire foot to contact the ground during walking, standing, or weight-bearing activities [1]. The assessment methods used, such as the LAA and navicular drop sign, in our study showed low values of navicular angle and height, indicating the presence of pes planus.

The dysfunction of the arch complex in pes planus is believed to alter the biomechanics of the lower limbs and lumbar spine [27]. We focused on evaluating spinal deviations using a scoliometer and the Trendelenburg test. No significant correlation was found between scoliosis and pes planus in this adolescent group, indicating that flat feet do not notably contribute to spinal deformities during this developmental phase.

Additionally, the lack of differences in physical activity levels between participants with and without pes planus indicates that flat feet do not immediately limit participation in daily physical activities during adolescence [28]. However, potential impacts on athletic performance, muscular endurance, and quality of life merit further investigation through ongoing longitudinal and functional studies in our population, as studies have demonstrated differences in performance [29].

Since pes planus is a foot deformity, the treatment of paediatric flat feet remains a topic of clinical debate, with the literature lacking clear guidelines on which paediatric patients need intervention and the overall effectiveness of such treatments. Nonetheless, emerging evidence supports the use of conservative, non-surgical interventions such as foot orthoses and physiotherapy, as potentially beneficial for specific patient groups [30]. The primary therapeutic goal is not necessarily the permanent correction of foot and ankle morphology but rather the reduction of deformity progression and the prevention of chronic, secondary complications throughout the kinetic chain [12].

This study provides important epidemiological data on the prevalence of pes planus and its related factors among Albanian adolescents, filling a notable gap in current research. These findings could inform local screening programmes and help develop preventive and treatment strategies customised for this population.

One limitation of the study is that we did not utilise podoscopes for screening or other advanced imaging techniques (such as 2D/3D computer systems or laser scanners), which offer detailed morphological assessments, especially of the subtalar joint and possible talocalcaneal coalitions [7]. Nevertheless, the LAA has proven to be a reliable and practical clinical measure for screening pes planus in a school setting, acting as a non-invasive and cost-effective tool for large-scale epidemiological and screening programmes.

Our study aimed to assess the prevalence of pes planus; however, as a cross-sectional study, it cannot determine the causal factors underlying this condition. Future research focusing on adolescents with pes planus could investigate anatomical abnormalities or other contributing factors associated with its development in this population.

## Conclusions

Pes planus is a common postural condition among Albanian adolescents, affecting nearly a quarter of secondary school pupils. The condition is significantly more common in males, while no strong links were found between pes planus and body mass index, body height, or spinal deformities such as scoliosis.

Given the potential of pes planus to cause long-term musculoskeletal problems, it is advised that routine screening programmes be introduced in schools. Early detection of pes planus through simple clinical assessments, such as the LAA and Navicular Drop Test, is essential, allowing for prompt intervention and possibly reducing the long-term public health impact of musculoskeletal disorders.

## Appendices

Student physical activity and sports participation

This survey is anonymous. Please answer each question honestly. There are no right or wrong answers.

Section A. Demographics

1. Age: ________ 2. Gender: ☐ Male  ☐ Female  ☐ Other  ☐ Prefer not to say 3. Grade/Class: ____________________ 4. School: _________________________

Section B. Physical Activity and Sports Participation

5. In the past 7 days, how many days did you do sports or physical activities (outside school Physical Education) that made you breathe harder or sweat? ☐ 0 ☐ 1 ☐ 2 ☐ 3 ☐ 4 ☐ 5 ☐ 6 ☐ 7 6. On those days, how much time did you usually spend doing these activities? _________ minutes per day (please, fill the number of minutes/day) 7. How often do you walk or cycle to and from school? ☐ Never ☐ 1-2 days/week ☐ 3-4 days/week ☐ Every day 8. If yes, how much time do you usually spend walking or cycling to and from school? _________ minutes per day (please, fill the number of minutes/day) 9. Do you spend time in outdoor play or helping at home (like chores, gardening, etc.)? ☐ Yes ☐ No 10. If yes, how often do you do these activities? ☐ 1-2 days/week ☐ 3-4 days/week ☐ Every day 11. On those days, how much time did you usually spend doing these activities? _________ minutes per day (please, fill the number of minutes/daily)

Thank you for participating in this survey!
